# Views of health system policymakers on the role of research in health policymaking in Israel

**DOI:** 10.1186/s13584-016-0088-1

**Published:** 2016-06-20

**Authors:** Moriah E. Ellen, Einav Horowitz, Sharona Vaknin, John N. Lavis

**Affiliations:** Jerusalem College of Technology, Ha-Va’ad ha-Le’umi St 21, Jerusalem, 93721 Israel; Israeli Center for Technology Assessment in Health Care, Gertner Institute, Chaim Sheba Medical Center, Tel Hashomer, 52621 Israel; McMaster Health Forum, McMaster University, 1280 Main Street West, Hamilton, ON L8S 4L6 Canada; Institute of Health Policy, Management and Evaluation, University of Toronto, 4th Floor, 155 College St, Toronto, ON M5T 3M6 Canada; Department of Clinical Epidemiology and Biostatistics, McMaster University, 1280 Main Street West, CRL 209, Hamilton, ON L8S 4K1 Canada; Department of Political Science, McMaster University, 1280 Main Street West, CRL 209, Hamilton, ON L8S 4K1 Canada; Centre for Health Economics and Policy Analysis, McMaster University, 1280 Main Street West, CRL 209, Hamilton, ON L8S 4K1 Canada; Department of Global Health and Population, Harvard School of Public Health, Boston, 677 Huntington Ave, Boston, MA 02115-6018 USA

**Keywords:** Knowledge transfer and exchange, Policymaker, Evidence informed policymaking

## Abstract

**Background:**

The use of research evidence in health policymaking is an international challenge. Health systems, including that of Israel, are usually characterized by scarce resources and the necessity to make rapid policy decisions. Knowledge transfer and exchange (KTE) has emerged as a paradigm to start bridging the “know-do” gap. The purpose of this study was to explore the views of health system policymakers and senior executives involved in the policy development process in Israel regarding the role of health systems and policy research (HSPR) in health policymaking, the barriers and facilitators to the use of evidence in the policymaking process, and suggestions for improving the use of HSPR in the policymaking process.

**Methods:**

A survey and an interview were verbally administered in a single face-to-face meeting with health system policymakers and senior executives involved in the policy development process in Israel. The data collection period was from July to October 2014. The potential participants included members of Knesset, officials from Israel’s Ministry of Health, Ministry of Finance, health services organizations, and other stakeholder organizations (i.e., National Insurance Institute). The close-ended questions were based on previous surveys that had been conducted in this field. Interviews were tape recorded and transcribed. Descriptive statistics were conducted for close ended survey-questions and thematic analysis was conducted for open-ended interview questions.

**Results:**

There were 32 participants in this study. Participants felt that the use of HSPR helps raise awareness on policy issues, yet the actual use of HSPR was hindered for many reasons. Facilitators do exist to support the use of HSPR in the policymaking process, such as a strong foundation of relationships between researchers and policymakers. However, many barriers exist such as the lack of relevance and timeliness of much of the currently available research to support decision-making and the paucity of funding to support research use. Suggestions to improve the use of HSPR focused on improving dissemination of research findings and ensuring that the research was more relevant and timely.

**Conclusions:**

This research demonstrated that health systems policymakers in Israel perceive having strong relationships and collaborations with researchers however there is room for improvement, e.g. partnering in research projects to ensure relevance and use. Furthermore, health system policymakers seem to be interested in receiving relevant research in a more useable format and are open to using research in decision making.

## Background

There is international awareness about the need to bridge the gap between research, practice and policy [1–6]. For close to a decade calls have been made to implement initiatives that link research to action. Knowledge transfer and exchange (KTE) has emerged as a paradigm to start bridging the “know-do” gap [7]. KTE is defined as “a dynamic and iterative process that includes synthesis, dissemination, exchange and ethically-sound application of knowledge to improve the health of [citizens], provide more effective health services and products and strengthen the health care system” [8]. While there are some excellent examples of effective links between research and decision making [9–11], there are still many challenges in using health systems and policy research to inform policy.

One of the main challenges that countries face in KTE is developing and using effective strategies to promote the use of research in policymaking [12, 13]. The two predominant actors in the KTE process are knowledge producers i.e. researchers and academics, and knowledge users i.e., managers and policymakers. Both knowledge producers and knowledge users have initiatives that they can undertake to support the use of research in policymaking. Knowledge producers produce the research, and if the research is not relevant, packaged in ways that highlight decision relevant information or readily accessible when decision makers need it, then the likelihood of decision makers utilizing the research evidence is poor [14–17]. Other barriers also exist to the use of research evidence in policymaking, for example academic research is frequently written in academic jargon and traditional scientific formats, and is not packaged or disseminated in a user-friendly manner [18, 19]. Numerous initiatives that researchers can undertake to support KTE have been proposed and studied, such as having researchers ‘push’ the research out through different channels, facilitating the use of research by ensuring that research is more readily available when it is needed and in a form that is easy to use, and utilizing knowledge brokers and other mechanisms to support linkage and exchange [20–22].

Knowledge users, such as policymakers, also have a role to play in the use of research. While research evidence may only be one of many factors within the decision and policy making process, there is an increasing awareness of its value [23]. Health system policymakers are required to make important and costly health system decisions related to the governance, financial, and delivery arrangements that determine whether the right programs, services and drugs get to those who need them [24]. Policymakers need to be open and receptive to evidence informed policymaking and they are integral to setting the climate for research use. Furthermore, if knowledge users do not know how to acquire, assess or apply the research evidence, and if they do not implement infrastructures that support the use of evidence, then the likelihood of using research evidence to inform policymaking will be poor. Lavis et al, and further reiterations of his framework [4, 20, 25], highlight some actions that policymakers can undertake in order to facilitate evidence informed policy making, such as training and implementing decision making processes that support the use of research evidence.

The success of any KTE strategy is dependent on tailoring the approach and initiatives that are implemented to the local context and the barriers and facilitators operating in these contexts [4, 25–27]. Understanding the local context and the views of the main actors in the process can assist in identifying the barriers and facilitators for KTE. Governments and international agencies provide recommendations and implement policies which they claim to be based on the best available evidence at the time. However, studies have shown that in many cases, these recommendations are not based on the best available evidence and there are gaps between the evidence that was available at the time a recommendation was made and the recommended action [28, 29].

While research in the area of KTE and understanding the views of key actors in the process and actual use of research in policymaking has been conducted in Canada, some Arabic countries, and elsewhere, [3, 13, 23, 30, 31], minimal work has been done in this area in Israel. The use of research evidence in health policymaking is an international challenge. Health systems, including that of Israel, are usually characterized by scarce resources and the necessity to make rapid policy decisions. The Israeli health system is based on the national health insurance (NHI) law, instituted in 1995, that provides every Israeli resident with a basic package of health care, i.e. universal health coverage. The main components of the Israeli health system are the Ministry of Health (MOH), four health insurance funds and other non-profit organizations. The MOH has overall responsibility for the health of the population and the effective functioning of the health care system. The system is financed through taxation linked to income and accounts for approximately 8 % of the GDP [32, 33]. In Israel, the high level policy makers (e.g., the Minister of Health) change frequently and may be limited in their knowledge or experience with certain health policy and system issues. However, those that support policymakers and senior executives involved in the policy development process in Israel, have the organizational memory, remain in their post for longer periods of time, and can support KTE initiatives and incorporate HSPR to inform policy. Due to the fact that policymakers are changing often, the culture and processes need to be implemented to support KTE and the approach to KTE and HSPR needs to be suitable to both policymakers, those that support policymakers, and senior executives involved in the policy development process in Israel.

Additionally, Israel is a small country with a lot of internal diversity. This means that there may be difficulty in conducting research on policy-type interventions since there can be a problem with the small “sample size” and generalizability. In addition, Israel faces diseconomies of scale; it has just as many national policy issues as much larger countries, but far fewer researchers to study these issues. Thus, it is even more important for researchers and policy makers to be more coordinated, and also be able to use research from other countries as a basis, that can then be assessed, adapted and applied to the Israeli context.

Previous research has been published that examines the experiences related to KTE in Israel of researchers who have conducted health systems and policy research in Israel. Most researchers that conduct health systems research in Israel are based either in research institutes, academic institutions, government agencies, the four health insurance funds, or hospital settings, and many researchers have cross appointments in a number of the aforementioned institutions. This previous research has demonstrated that less than half of the respondents were involved in various KTE activities such as interacting with knowledge users throughout the research process and developing reports and summaries that used language appropriate for their target audiences [34]. More than a third of the researchers in Israel reported that they were frequently or always involved in interactions with target audience through the research process (i.e. during developing a research question or executing the research) or through formal or informal meetings during conferences, workshops or conversations. However, less than half of the researchers stated that they were engaged in bridging activities aimed to facilitate target audiences to use research [34]. While there is engagement from the researchers in Israel, it is important to note that health system researchers are only one part of the equation when it comes to ensuring the use of research in health policymaking. Policymakers in the health system also play an important role in the use of research evidence to inform their decisions. Previous research in Israel has documented the reforms made to the health system as well as the potential role of data to inform policymaking. While the research did demonstrate that data use increased over time and decision makers did rely on data, at times key data were missing and furthermore, the policymakers rarely explored in a systematic way how data could contribute to the decisions they face. In addition, one study found that even where the intention may be to use data to inform policymaking, political motives are also a strong force [35, 36]. The purpose of this research was to explore the views of health system policymakers and senior executives involved in the policy development process in Israel regarding the role of health systems and policy research (HSPR) in health policymaking, the barriers and facilitators to the use of evidence in the policymaking process, and suggestions for improving the use of HSPR in the policymaking process.

## Methods

A survey and an interview were verbally administered in a single face-to-face meeting with health system policymakers and senior executives involved in the policy development process in Israel policymakers in Israel.

### Study population

The survey population consisted of Israeli health services policy makers. This definition included those who are involved directly in health policy making as well as those who support these processes (i.e.: those who consult with policy makers or support them with relevant information such as CEOs, managers or heads of different governmental departments and councils). Individuals who fit these criteria and who were involved in the last five years in at least one health policymaking process in the Israeli health system, were eligible to participate.

### Selecting the sample

It was estimated that there are approximately 60 individuals who can be considered to be health services policymakers or in support roles for these policymakers in Israel. Therefore it was decided that all potential participants will be invited to take part in the survey. The list of potential participants was identified through four main avenues: 1) searching publicly available web sites (e.g.: Israel’s Ministry of Health) to identify individuals in appropriate positions, 2) consulting with leaders in the health policy arena who are familiar with the health policy making landscape in Israel, 3) contacting the National Institute for Health Policy to determine if they could identify additional participants, and finally, 4) using a respondent-driven sampling technique (i.e., asking participants at the completion of the interview if they can identify any additional participants that may have relevant information for our study). The sampling frame was purposefully broad to ensure that all potential participants were captured: therefore the survey may have been applicable to a smaller number of participants than what was sent out.

The final list of potential participants included officials from the following: the Knesset, Israel’s Ministry of Health (CEO, heads of divisions and departments, deputy directors and heads of national councils); Ministry of Finance; health services organizations (CEOs and vice presidents); and other organizations (medical centers Hospitals CEOs, National Insurance Institute of Israel’s CEO, Israel Medical Association; members of the Knesset).

### Developing the survey and interview guide

The survey was based on a survey that has been tested and has shown high internal consistency and good face and content validity [37], which itself formed the basis of other surveys that have been conducted in this field [3, 30]. However, this version was lengthy and was therefore modified and adapted to the Israeli context. The survey tool focused on: a) factors that have an effect on the health policy making process, b) barriers to use HSPR by policy makers, c) linkage and exchange activities between researchers and decision makers/policy makers, and d) views on KTE [3, 13]. The surveys consisted of a demographics section and closed ended questions, with response options on quantitative scales. The response options to the quantitative scales were given on ordinal scale ranging from 1 to 5 (were: 1 = “strongly disagree” and 5 = “strongly agree”) with a middle neutral category (3 = “neither agree nor disagree”). The interviews consisted of open ended questions that focused on the barriers and facilitators to using evidence to inform policymaking and suggestions to improve the usage of HSPR as part of the policy making process.

The survey and interview tools were translated into Hebrew using the process recommended by the World Health Organization [38]. First, the tools were forward translated to Hebrew by a researcher familiar with the terminology of the area covered by the survey and interview. Second, the tools were back translated to English by an independent translator whose mother tongue is English. Third, both versions (English and Hebrew) were given to a bilingual expert in order to resolve the inadequate expressions/concepts of the translation. Finally, the Hebrew translation was corrected to its final version.

### Recruiting the sample and administering the survey and interview

An initial email, in Hebrew, was sent out to all potential participants inviting them to participate in the study. The letter described the purpose of the study and offered the recipients the opportunity to participate voluntarily in the study and contribute based on their experience. Recipients were also told that the survey and interview can take place at their work place or another location that is convenient for them. A second reminder was sent two weeks after the initial request, followed by a third reminder which was sent a month later. Those who did not respond to the third reminder were contacted by phone.

Both the survey and the interview were conducted in a single face-to-face meeting and the latter was recorded and transcribed. The interviews took place at the location most convenient for the participants, which most often was their workplace. Participants were asked to sign a consent form and were assured that all responses would be anonymized. The data collection period was approximately four months (July to October 2014).

### Data analysis

All the quantitative responses were exported to the Statistical Package for Social Sciences (SPSS) and analyzed using descriptive statistics. Descriptive analyses were conducted for closed-ended questions. For close-ended questions about views on the barriers and facilitators to use HSPR we combined the two highest response options.

The interview data were analysed using a constant comparative method for the thematic analysis. Two researchers independently read and coded the transcripts. First we read the entire interview transcript to get a sense of the whole interview and initial impressions. Then we read the text a second time, coded units of text, and compared initial codes. Coded segments were then re-analysed, coded into subcategories, and compared again.

The study received ethics exemption from the Jerusalem College of Technology’s ethics committee.

## Results

A total of 73 potential respondents were contacted; three of them did not meet the criteria for participation in the study. 32 policy makers were surveyed and interviewed (response rate of 46 %). Non-response was due to the following reasons: 4 refused, 26 did not respond to our initial contact and subsequent letters, and 8 potential respondents agreed to participate but could not commit to an interview during the time frame of study. Among the 32 respondents, 23 were males and 9 females. The average age of the respondents was 54.7 (SD 11.3) years. The age range was 34 to 83 years. The response rate was higher for respondents working for government than for other organizations and the response rate was lower for the ministers, director-generals and CEOs than for other positions. The set of respondents has a greater concentration of government employees, and a lower concetration of top-ranking officials than did the study population. 56 % (18) of participants were from the Ministry of Health, 12.5 % (4) each from health service organizations and national councils, and the remainder from other organizations such as hospitals, the national insurance institute, the Israel Medical Association, the Ministry of Finance and members of the Knesset. 16 % (5) of the respondents were either Ministers, current or past director-generals of the ministry of health, or CEOs, 28 % (9) of the respondents were deputy director-generals or vice-presidents, and 56 % (18) were in other positions i.e., department heads or chairpersons of national committees (see Table 1).

**Table 1:** Composition of the set of potential respondents compared to the actual respondents by organization type and level in the organization

	Potential Respondents (n)	Actual Respondents (n)	Response Rate (%)
Organization Type			
Government (MOH, MOF, etc.)	39	20	51 %
Non-government	34	12	35 %
Level in the organization			
Ministers, director-generals, CEOs	18	5	28 %
Deputy director-generals/VPs	17	9	53 %
Others (includes department heads, chairpersons of national committees and other)	38	18	47 %
Total	73	32	44 %

### Quantitative analysis of survey questions

#### Views on the role of HSPR in influencing the health policymaking process

Respondents indicated that there are both significant barriers and important facilitators in the real-world application of HSPR in Israel. Most respondents felt that the use of HSPR was hindered due to practical constraints such as financial implications and just over half felt that it was hindered due to politically sensitive findings (Table 2). About two thirds of respondents agreed that evidence from HSPR does help policymakers identify and/or choose policy alternatives, which reflects and implies an actual use of research, and close to half of the participants stated that evidence from HSPR does help raise awareness on policy issues. Yet, only a quarter of participants felt that evidence was presented to them in a timely manner or in a format that was easily understandable.

**Table 2:** The role of HSPR and the factors that influence the use of HSPR by health policymakers and stakeholders Israel

	Percentage Agree or Strongly Agree
Use of evidence from HSPR in policy was hindered by practical constraints to implementation such as financial implications	91
Evidence from HSPR does help health policy makers and stakeholders to identify and/or choose policy alternatives	63
Use of evidence from HSPR in policy was hindered by findings that were politically sensitive or were inconsistent with a policy direction	52
Evidence from HSPR does help raise health policy makers and stakeholders’ awareness on policy issues	49
Use of evidence from HSPR in policy was hindered by a non- receptive policy environment	34
Lack of coordination between policy makers and researchers hindered the use of evidence from HSPR in the health policymaking process	32
Evidence from HSPR was presented to policy makers and stakeholders in a timely manner and in a format that they can understand	25

#### Views on the barriers and facilitators for KTE activities

More than two thirds of the participants felt that the national funding organizations formulated their funding calls in response to regional and national needs and that national funding sources support KTE activities (Table 3). Most participants also agreed that there were structures and processes in place to link them with researchers and that they had the necessary skills to acquire, assess, adapt and apply the relevant research. Less than half of the participants felt that there were significant barriers in place to prevent the use of HSPR. Less than half of respondents felt that organizations that conduct HSPR assisted with KTE activities by making financial and human resources available to assist in the transfer and exchange of knowledge (Table 4). Less than half of the participants felt that the currently available research aligns with the needs of the knowledge users and that the currently available research aligned with the country’s priorities (Table 5).

**Table 3:** Potential facilitators and barriers to the use and implementation and use of KTE activities

	Percentage Agree or Strongly Agree
Facilitators:	
National funders formulate their priorities and calls for proposals in response to national and regional needs.	78
National funding sources encourage KTE activities.	69
Structures and processes exist to link you with researchers	68
Policymakers have access to technical support for acquiring, assessing, and applying HSPR research	68
Funding sources (e.g., granting agencies) consider KTE activities an allowable expense	65
Personal and organizational contacts among policymakers and researchers were quite stable over time	61
Policymakers create opportunities to develop joint HSPR research initiatives with them	45
Policymakers invest financial and/or human resources in joint HSPR research initiatives with them	45
Policymakers invest financial and/or human resources in KTE activities	42
Barriers:	
Priorities in the health system draw attention away from HSPR research	43
Policymakers lack the expertise for acquiring, assessing, and applying HSPR research	31
Policymakers do not make decisions on the basis of HSPR research	24
Policymakers do not have technical access (i.e. journal subscriptions, links to research) to the appropriate databases to search for HSPR research	10

**Table 4:** Additional facilitators and barriers at the level of organizational support for KTE activities

	Percentage Agree or Strongly Agree
Organizations that conduct HSPR made available financial and human resources to assist with KT activities	46
KT was hampered by a lack of incentives for KT activities within organization’s that conduct HSPR	15
Organizations that conduct HSPR were not seen as a credible source of research	7

**Table 5:** Alignment of available research to needs of knowledge users

	Percentage Agree or Strongly Agree
Available research coincided with my country’s priorities (e.g., with a National Research Agenda)	48
Available research coincided with the needs and expectations of target audiences	37
Available research was not considered relevant by policymakers	11
No research was ready for use	4
Available research lacked credibility among target audiences	0

#### Views of what influences the health policymaking process in Israel

Most respondents felt that broad challenges in intergovernmental (i.e. Ministry of Health, Ministry of Finance) relations hindered the health policymaking process and more than half felt that broad challenges in government/provider relations hindered the health policymaking process (Table 6). The four main influencing factors that were perceived to have exerted the strongest influence on the health policymaking process were limited health funding, health insurance funds, the media, and physician associations (Table 7).

**Table 6:** Factors that influence health policymaking in Israel

	Percentage Agree or Strongly Agree
Broad challenges in intergovernmental (i.e. Ministry of health, Ministry of Finance) relations hindered the health policymaking process.	91
Broad challenges in government/provider relations hindered the health policymaking process.	59
Policy formulation is usually based on internal Ministry of Health discussions and ad hoc process rather than evidence based processes	34

**Table 7:** Groups or factors that exert a strong influence on the health policymaking process

	Percentage Agree or Strongly Agree
Limited health funding (the economy)	100
Health insurance funds	77
Media	71
Physician associations	59
Values of governing parties	41
Public opinion	38
Other countries’ health policies	36
Nursing associations	30
Other types of health professional associations	20
Research about problems related to healthcare or health systems	19
Donor organizations	3

### Qualitative analysis of interview questions

#### Barriers and facilitators to the use of HSPR in policymaking

The main barrier that was mentioned by all participants related to the lack of timeliness, dissemination, and relevance of the research (Table 8). With respect to the dissemination of the research, all participants felt that the research was often not timely and therefore it was frequently not relevant i.e., “there is a kind of delay between the decision making and when you get… the evidence sometimes comes too late, so it, at most, confirms the decisions you already made, but many times there is nothing to rely on”. This could be either because of the time it takes to conduct the research, the speed with which decisions need to be made, or the delay between conducting the research and making the research publicly available.

**Table 8:** Representative quotes on the barriers to the use of HSPR in health policymaking/decision making in Israel^a^

Theme	Representative quotes
1. Barriers related to the actual research and dissemination of the research	
• The research is not timely	The time from when the study was conducted until it was published. This is a general problem with research – it takes too long until they are published and it is unclear that the data is still relevant to current reality. One of the problems is that it is very rare that you have the information you want. The problem is you want to get information, information that you don’t have, and do not have time to wait for it. Most of the studies that I see come after the fact, and this is 20/20 hindsight.
• The research is not always relevant i.e. the research question does not match the need	[Researchers] don’t always ask us what we need to know, what are the issues that interest us, before planning the research. Then they come and say, “Use this,” but we do not need it.
• Research from other countries is not always applicable	Discrepancies between international research and international data and the situation in Israel Doubts about the relevance of studies and data from overseas to the unique situation in this country, requires self-examination, [that] cannot always be done.
• Concerns regarding the type of research and the quality of research	Research where it is not clear what was their methodology… I want to see the methodology of the study to see how much I can trust it, critically, and if I do not have access to the methodology, it makes it difficult for me.
• Dissemination of the research results	Studies get published but they remain at the level of articles and conferences, but they don’t break down into the particulars to examine applicability.
2. The ability to make the change in the organization	We have a highly structured health system with a particular structure where it is not always easy to implement, to fit some things to the evidence, in a framework where it’s very difficult to make changes in the structure of our system.
3. Interests from different stakeholders (including political agenda)	There are all sorts of considerations for the HMOs in implementing… especially of the doctors and, definitely, of the government. Barriers of personal views, of politics, the media, the pressures from voters, the wealthy. Some will adopt this wholeheartedly. If it fits with your doctrine, then it’s very good to come out and say “I just happened to find …”.
4. Policymakers’ preconceived notions regarding decision making and attitudes towards research	People have preconceptions about what should be done, no matter what the study shows. The main problem is that policy makers in Israel do not want to make decisions based on data, and certainly in cases where the data do not support their gut feelings, their ideology, their tradition … don’t want to hear, don’t want to implement, don’t want to internalize it.
5. Policymakers’ understanding of the research	Many of the policy makers do not know how to read research … social research, which is what create policies, add other variables to the picture, variables that doctors don’t have a clue. They sometimes do not even realize their importance.

^a^The themes in the table are presented from the most to the least common themes mentioned in the interviews

With respect to the actual research, most participants felt that at times, the research was not relevant and did not match the needs of policymakers i.e., “the research does not always answer the question exactly. I mean, it is broader or narrower, it looks from another angle, so it does not always answer the question that arises at the moment.” Furthermore, participants felt that many times while there may be research that answers similar questions from other countries, it was not relevant for the Israeli context since Israel’s climate, context and culture is different and therefore other country’s research cannot easily be adapted. For example, one participant stated that it’s “very difficult to rely on policy studies from countries that have a completely different health system […]. So the adaptations we need to make are very complex, which makes the research not always relevant.” Many respondents also cited the typically ineffective dissemination methods as a barrier, either in the packaging (i.e., “Sometimes we need a bottom line … So what are the conclusions and what are the recommendations”) or the channel (i.e. academic journals) that the research is disseminated. Another barrier that was mentioned many times was the logistical limitation or inability to make the changes in the organizations.

Some participants felt that the positions taken by different stakeholder groups, such as industry, and the ‘political agenda’ were barriers. It was felt that “if research does not fit with their (policymakers) perception, so they ignore it”. Other participants also felt that policymakers’ pre-conceived notions regarding the decision making process and the inclusion of research in that process were major barriers to the use of HSPR in decision making. Some participants felt that “policymakers [think they] know everything” and that it has become the norm for decisions to be “based on personal preferences and on the basis of subjective understanding of reality, this is the tradition that exists in Israel.”

Finally, it was felt that at times, many different individuals are working on the same issue in such a small country at the same time and there is a “situation in which a lot of people are working on the same thing in parallel without interaction – there is no communication within the Ministry of Health, outside the Ministry of Health, in the HMOs themselves. And that, I would not say “full gas in neutral” but why do we need to use four cars at the same time if you can put them all in the same car so they can go together? Lack of communication between the relevant parties creates a situation where you are sometimes not aware of the existing policy- by the time you are aware of it, you suddenly discover that you are too late. So you say, “I was working on this, I tried to lead something, and in fact it already exists”. Because people forget they have to pass on the information on policies”. This lack of communication and alignment acts as a barrier to the use of the appropriate information when developing policies.

Most participants felt that the main facilitator to the use of research in policymaking and decision making was the collaboration and relationships between researchers and policymakers (Table 9). Different forms of collaboration were mentioned such as ensuring experts are included in the decision making process i.e., “It’s that there is a professional in this decision-making process that is critical. Decision-making is not carried out by those who don’t know how to read research.” Another form of collaboration was having round tables with the National Institute for Health Policy (NIHP) i.e., “Round tables at the healthcare system level that can be a place for raising needs and requirements. For example, meetings of the NIHP, which happens each year, where they collect number of issues and address them; and those who participate in it include academics, and policy makers, and officials from sick funds, so there is discussion and dialogue within the country.”

**Table 9:** Representative quotes on the facilitators to the use of HSPR in health policymaking/decision making in Israel^a^

Theme	Quotes to support
1. Collaboration and relationships between researchers and policymakers	A small country, where there are many connections between people, so they have many opportunities for sharing information and transferring ideas etc. That helps… Since there is the Knesset Research and Information Center, we have a better ground for work on, because the work is based on data and not only on intuition or biased knowledge that is only based on my life-experience, and I don’t know other people’s life experiences well enough. Combination of researchers who are also involved in clinical work, i.e. management, as well as researchers, who can influence research directions and then get results that fit their decision making.
2. Facilitators related to the actual research and dissemination of the research	
• Outlining the relevance of the study to Israel	Research that relates to the population in Israel is by nature better able to influence policy than general research … The more it refers to the Israeli population, or a specific sector where the question is, then its validity would be greater, it will have more weight.
• Type and quality of research	The quality of research – relevant research, done on a large scale, with the participation of relevant people with prestige and influence on decision makers. Research based on administrative data is very helpful to rely on, and not only on sample and survey data. The quality of research which is reflected … also the methodology, even where it was published.
3. Outside pressure	
• Public pressure	Understanding of the need by the public, so they can come out and demonstrate and influence decision-making
• Media pressure	An increase in the prevalence of the phenomenon as is reflected in the media. Politicians are very sensitive to the media …
4. Culture that supports the use of research in decision-making	The great openness and the desire of decision makers, and their understanding that such studies can be a working tool, or a tool that contributes to decision making. It’s this willingness of the decision-makers

^a^The themes in the table are presented from the most to the least common themes mentioned in the interviews

The next main facilitator that was mentioned was related to the actual research such as ensuring that the research outlines the relevance of the study results to Israel i.e., “Research that relates to the population in Israel is by nature better able to influence policy than general research.” Outside pressure from both the public and the media was also viewed as a facilitator to support the use of research in decision making. Some respondents felt that a strong facilitator to the use of research in decision making is the existing culture and that there is a “general approach to try to base decisions on facts.” This could be because some decision makers have a background in research and they institute this type of culture in their departments i.e., “People who came from research … and create a culture of a kind of decision-making in the offices.” Another reason for the existence of this culture could be a spill over effect from medicine i.e., there is an accepted culture in medicine of evidence based practice and therefore there is an overall push that all decisions should be based on research i.e., “the medical culture – they use a lot of evidence based. There is a push for research …”.

#### Main KTE activities in Israel

All participants mentioned attending both local and international conferences as the main KTE activity in Israel as they serve as a basis for collaboration and future research (Table 10). Most participants mentioned building formal relationships between researchers and policymakers as an important KTE activity in Israel. This included being partners in research production or having formal meetings and collaborations to discuss research i.e., “All the relationships we have with the research institute, which is expressed by raising issues.” Another KTE activity that was mentioned was collaboration between researchers and decision makers on committees for specific health system issues such as i.e., wait times and quality improvement.

**Table 10:** Representative quotes of main KTE activities in Israel^a^

Theme	Quotes to support the theme
1. Attending local and international conferences	The presence at and support of conferences, which I think are also a good tool to design future research. Then you report what you have done, but it’s actually the infrastructure for the things that follow.
2. Building *formal* relationships between researchers and policymakers	
• Partners in research production	We’re constantly involved in research … all kinds of researchers come to us.
• Meeting to discuss research	The activities of the Ministry of Health (MOH) Management with the Gertner Institute(Institute for Epidemiology and Health Policy Research), - periodic meetings to talk about research and what are the needs of the MOH, and what Gertner can give.
3. Collaborating on committees for specific issues	We direct advisory committees in many fields to define quality indicators in hospitals in Israel and carry out studies of clinical outcomes, form partnerships with relevant scientific unions, and collaborate in studies they do and conduct our own studies… for formulating policy, eventually.
4. Linkages with international organizations i.e. OECD	The MoH representative in the OECD makes the integration of what is happening with our hospital quality indicators and what is done with quality indicators around the world and we participate in international studies as part of the OECD countries to make international comparisons. Meetings with international bodies such as the OECD, the World Health Organization, conferences of all sorts.

^a^The themes in the table are presented from the most to the least common themes mentioned in the interviews

#### Suggestions to improve the use of HSPR in health policymaking in Israel

Most participants felt that in order to improve the use of HSPR in policymaking in Israel, the dissemination of research findings needs to be more effective i.e., “to reach politicians – in order to speak the same language, they need to get it in some kind of a “nutshell”, so that they understand the significance and importance …where it affects them, what benefit it gives them” (Table 11). The research has to be delivered to the policymakers in a manner that is easy to understand and apply. Some respondents recognized that the responsibility falls on both knowledge producers and knowledge users i.e., “Concerning the transfer of information – it’s true not only on the side that posts the information, but also on the receiving side. That decision-makers have someone in charge of interactions.”

**Table 11:** Suggestions to improve the use of HSPR in health policymaking in Israel^a^

Topic	Quotes to support
1. Increased and more effective dissemination of research findings to policymakers	There should be someone who connects and create this link between policymakers and researchers, because, in this era of profusion of studies in various fields, we need to have someone to do the integration before they submit it to policy makers. ᅟ You come to the decision maker and show him a study and he has no idea how to judge it, how reliable it is, how valid it is, whether the methodology is correct … ᅟ Need to find form of transferring information that is convenient to policy makers.
Practical ideas re: implementation	*Consolidating dissemination of findings:* Once the institute has published something, send it, maybe not every month, but maybe several times a year, every quarter … you can send abstracts … and suddenly I see that it’s something very interesting to me and that I’m working on at the moment. I may not read it now, maybe I’ll print it and read it later… ᅟ Some kind of a digest, a site that sends abstracts directed at policy issues, that I can choose from a list of topics. ᅟ A quarterly, bi-annual or annual publication that collects all the articles… Whoever wrote an article and thought it is relevant to the field of policy should send it is to this place and they can distribute it as a news-letter once every X time, and you can see all the things that were published, and see if you want to get into it or not. Not every time there is a new study- not something that overwhelm people- but something they know that comes out between 1 and 4 times a year, each time an email. And whoever wants to, can look at it. ᅟ *Disseminating concise summaries of study findings:* Distributing very concise summaries by e-mail to the target audience. If it’s really an abstract- 3-5 lines- I can read it. ᅟ *Inviting researchers to present in the decision-makers’ organizations* Inviting researchers to present their research work within a policy-oriented framework, i.e. to policy makers within the organization, not in an external conference … for example, a board meeting of the Ministry of Health or our meetings, if a researcher can came to present his work more frequently. ᅟ *Training both knowledge producers and knowledge users on effective dissemination and usage of research findings:* To train policymakers in using findings efficiently and to train the reviewers on how to correctly present and how to fit the needs of policy makers in the way they pass on the information. ᅟ *Pushing findings:* Researcher who conduct a study and has findings should push it. Publish it in all sorts of ways. There are many ways to publish. Pushing knowledge – but pushing is something you do again and again. You sent something and there is no response, you can send it again. Don’t be afraid to push, to be a little aggressive in pushing knowledge that seems very important. To push it via e-mail or other kinds of mail, or request an appointment or send it again, or remind them, if the situation arise. Marketing the information, the knowledge, upwards and onwards. ᅟ *Using knowledge brokers or leaders to disseminate the information:* Research should involve people of reputation and status that may promote this tool called research to decision makers, including knowledge brokers. ᅟ *Enabling the ability to search in national language* i.e. *Hebrew* A search engine in Hebrew- that I can have one in Hebrew as well, some place that collects all the studies and then I can search … something more accessible. It does not have to be only peer-reviewed studies, but also documents from Brookdale (Centre for Health Policy Research) and the like…
2. Collaboration between researchers and policymakers on research production	At the stage when they develop research, that it should kind of fit the needs, i.e. to be involved early in the development of research so that it would answer my needs, that someone will take into account issues and methodology that interests me. The researchers will know what is the product that I need. ᅟ Building research together with policymakers, in advance- defining the main objective, reaching consensus on its meaning..something that is done in advance together. ᅟ Interactions with policy makers in advance to define the questions that interest them and are still unanswered by research. In major issues, you should also consult with them on study design, in a way that will make it more relevant to policymakers.
3. Opportunities for official linkage and exchange between policymakers and researchers such as;	If you define in advance the role of decision maker, or an executive; if you make it a part of his roles not only to provide services but also to create an interface with the Academy. So you generate, in advance, an organizational commitment, even a physical one, for the purpose of learning, reading, hearing and cooperation. .
• Conferences	Periodic meetings with the NIHP. The Health Ministry has a level that knows what is happening at the NIHP. At my level, which is an intermediate level, we don’t know… If there were conferences to the middle levels… to see what you are researching, what you are doing, how it relates. ᅟ For me, this whole story of conferences and journal clubs is very helpful, but many policy makers don’t see its importance of it and make time.
• Journal club/research clubs	Periodic meetings – invite various organizations to present their fields of interest to the Health Ministry, the HOMs management, all kinds of decision-makers. There are institutions such as Tel Aviv University, Brookdale (Centre for Health Policy Research), Gertner (Institute for Epidemiology and Health Policy Research), places like this, that can, once in a while, come and present their work in these fields and to see what are the relevant components and where they can augment each other. ᅟ Formal meetings. To make some seminar or a consensus conference where policy makers will be invited (usually there are only researchers in such consensus conferences) so a kind of sharing, joint seminar....
4. Increased budget to support KTE	Instead of investing 100 NIS in research, spend 90 on research and 10 on implementation…

^a^The themes in the table are presented from the most common themes to the least common themes mentioned in the interviews

Practical suggestions were provided regarding how to improve the dissemination of research findings i.e.,

- Consolidating the dissemination of findings and either
  - o developing a website focused on health research that will send emails with list of recent research, associated abstracts and links to the research,
  - o compiling a quarterly newsletter that highlights recent relevant research, and/or
  - o providing short summaries of research, not more than 3-5 lines
- Sending concise, targeted emails with links to the research,
- Inviting researchers to present in the decision-makers’ organizations
- Training both knowledge producers and knowledge users on effective dissemination and usage of research findings
- Using knowledge brokers or leaders to disseminate the information
- Enabling the ability to search in the national language i.e. Hebrew

Most participants also stated that increased collaboration between researchers and policymakers on research production can ensure the use of research in decision making i.e. “The academy cannot be completely cut off, if it wants to influence, if it wants to be a part of things, it needs to connect with the decision making process at an earlier stage.” Furthermore, providing opportunities for official linkage and exchange between policymakers and researchers can improve the use of research in decision making. These opportunities can occur either at conferences or through research meetings.

However, some participants felt that conferences were not so useful because they are very subject specific, limit the number of participants, and are not accessible to all due to the cost i.e. “At the Dead Sea Conference, for example, they limit both to a specific subject and also to a very small number of people” and “Making it so that the conferences are not so expensive … or open them, extend more invitations to conferences.” Furthermore, some participants felt that conferences were not so useful i.e. “I don’t use “they said so at a conference” when I make a policy decision.” Additionally, conferences should not be viewed as ‘the tool’ to disseminate the knowledge since many times they key knowledge users are not present i.e., “Usually the Director General and Deputy Director General and the Minister come, greet and leave. Everyone knows it… How come researchers do not realize that this will not lead to results if the person who needs to hear isn’t there … I’m not saying we should stop having conferences, but it can’t be the tool (8(”.

## Discussion

In this study, we investigated the views of health system policymakers’ and senior executives involved in the policy development process in Israel on the use of HSPR to inform decision making. Both the quantitative and the qualitative components of the study demonstrated that while there are many barriers in place, there are numerous facilitators that are already in place and support evidence informed policymaking and they can also be capitalized on for future initiatives. The barriers primarily focused on the currently available research and the lack of some of its relevance and timeliness to support decision making, the usually ineffective dissemination of research, and interests of different stakeholder groups. The main facilitator that was identified both in the quantitative and the qualitative research was the strong foundation of relationships and collaborations between researchers and policymakers or decision makers. Participants provided a wealth of suggestions regarding how to improve the use of HSPR to support health policymaking and decision making.

The diversity of respondents can be viewed as both a strength and a limitation of the study- it provides us with a wider context of many of the actors in the policy making process, but, on the other hand, their divergent perspectives and personal experiences can lead to different opinions on the use of HSPR and its role in the policymaking process, thus obfuscating the results. While we may note the differences in response rates for each category of respondents, two issues must be taken into consideration. Firstly, the number of potential respondents in each category is relatively small, especially if we integrate the different categories together (i.e. director level- government, other-non-government, etc.) as well as other subdivisions within each category, so that it is likely that potential systematic biases may be overshadowed by the individuality of each respondent’s unique position. Secondly, the categorizations was based on the respondents current position, or the one that they held within the previous 5 years- many of the respondents have a long history of different positions within the healthcare system or the policy-making process, therefore their response may reflect experiences and perspectives that place them into more than one category.

The biggest strength of this study is that, to our knowledge, this is the first study examining the views on the use of HSPR by health system policymakers and and senior executives involved in the policy development process in Israel. Further strengths include that the close-ended questions were built on a pre-existing and validated instrument and that the survey was administered face-to-face as opposed to online, thus promoting a somewhat higher response rate. However, our survey is not without limitations. The two main limitations are: a) despite the fact that the researchers went to great lengths and repeated attempts to recruit participants, the response rate is lower than hoped and b) the survey is based on self-reports and therefore, social desirability bias cannot be excluded. These limitations may influence both internal validity and the generalizability of the findings to the broader population.

Another limitation of the study is that it asked respondents to relate to the research-policy interface overall. It is quite likely that the effectiveness of the interface varies across institutions, policy area, type of research, etc. There may also have been some differences in among various types of respondents in their perceptions; however given the relatively small sample size it is not possible to differentiate the effects of those differences from just individual variability. There are important opportunities for further research to explore what accounts for the variation in the effectiveness interface in Israel; both quantitative studies and in-depth case studies can play an important role.

Some of the findings of this study are in alignment to the findings of other international studies. Similar to other studies, this study identified the need for the timeliness and relevance of the research as well as the local applicability of the research as factors that can influence the uptake of research evidence [15, 39–41]. Furthermore, numerous papers and studies have been written about the importance of relationships and collaboration between researchers and decision makers which can facilitate the increase of evidence informed policymaking. Barriers such as lack of personal contact and opportunities to discuss challenges and research opportunities between researchers and knowledge users impede the use of research in policymaking [15, 17, 39, 41–44]. The issues uncovered in this research, while not new in the field of KTE, are new to the Israeli context. These findings provide insight as to the challenges experienced in Israel and provide researchers and decision makers with the necessary evidence to build interventions to support the use of HSPR in policymaking and decision making.

There are some differences as well between this study and the international literature. One main difference is with respect to the groups or factors that exert a strong influence on the health policymaking process. In our study, the three biggest influencing factors were limited health funding, the health insurance funds and the media; however, in other studies, other factors were found to have a strong influence such as physician associations, donor organizations or the values of governing parties [13, 31]. Furthermore, in two other studies, about half to two-thirds of the respondents agreed or strongly agreed that research about problems related to healthcare or health systems exerted a strong influence on the policymaking process (46 % in the Eastern Mediterranean Region and 66 % in Canada; the Canadian study focused specifically on research related to healthcare providers), while in our study only 19 % agreed or strongly agreed with that statement [13, 31].

Also, based on the quantitative and the qualitative findings, it is apparent that there are strong relationships between researchers and policymakers in Israel. This is in contrast to the situation in many other countries where these relationships appear to be less well developed. For example, in one study from the Eastern Mediterranean region it was found that only 43 % of respondents agreed that there are contact and collaborative relationships between researchers and policymakers and/or decision makers [13]. The relatively strong relationships in Israel should be capitalized upon, as is elaborated below.

Numerous frameworks have been developed, describing an array of initiatives related to KTE, [25, 26, 45–50] yet many of them operate at the individual or clinical level and not at the country level. Lavis et al propose a framework to assist in assessing country level efforts related to KTE and provide insight as to which elements a health system should have in place in order to facilitate the use of research in policymaking [25]. There are seven main elements which are:

1. Climate for research use (i.e. ensuring a climate where researchers, policymakers, and decision makers value the use of research in policy and decision making)
2. Research production (i.e. ensuring appropriate capacity (i.e. financial and human) to conduct highly relevant research)
3. Push Efforts (i.e. efforts undertaken to push the applicable research out to potential users through appropriate channels)
4. Facilitating Pull Efforts (i.e. ensuring the necessary infrastructure and tools are in place so research can be readily and easily accessible)
5. Pull Efforts (i.e. efforts undertaken by users of research to acquire, assess, adapt and apply the appropriate research)
6. Linkage and exchange efforts (i.e. initiatives to create open relationships and dialogues between the research producers and the research users)
7. Evaluation efforts (i.e. evaluation of KT initiatives).

Based on this research, policymakers in Israel perceive that the linkage and exchange efforts exist in Israel and there is a strong foundation on which to build upon. Strong links between policymakers, stakeholders, and researchers can enhance the transfer of research into practice [51]. Linkage and exchange efforts fundamentally occur when there are positive relationships between research producers and knowledge users, which seems to be the case in Israel [52].

However, policymakers identified the biggest challenges to be within the research production and push efforts. With respect to research production, the quality of the research, its topical relevance, its operational usefulness, the solutions or recommendations associated with the research, and the credibility of the source are all important characteristics that can enhance the use of research in policymaking [48, 53, 54]. Health systems need to ensure that they have the capacity to conduct research and fund the creation of new knowledge [55]. Furthermore, changes are needed so that local stakeholders such as policymakers and decision makers can have influence in determining the nature, quality and applicability of the research being conducted [55]. Ownership of research and research ideas by stakeholders are also important [53]. If the Israeli health system wishes to support the creation of new knowledge that is applicable, future initiatives should focus on establishing regular priority setting processes with researchers, stakeholders, and policy makers, funding new research in the form of partnerships between researchers and knowledge users or health services agencies, and ensuring the overall capacity to conduct or commission research [20, 25, 56].

With respect to push efforts, ensuring the effective dissemination of research findings is essential. The push efforts that researchers or intermediary groups undertake can bring research evidence about an issue to the forefront and to the attention of policymakers and inform the policy development and implementation processes [12]. The messengers and the packaging of the research are important characteristics to consider [41, 53], and were identified by participants as interventions to consider in improving the dissemination of study findings. Traditionally, researchers disseminate their findings via publications and conferences; these are important initiatives but primarily contain the research findings within academic circles. ‘Pushing’ the knowledge out to users requires re-packaging of information and highlighting actionable, jargon-free messages [41]. To have an impact, research findings must be translated and adapted to specific contexts and situations [53]. Developing applied products and tools that help knowledge users see the relevance and usefulness of the research is a factor that can affect the successful transfer of research into practice [22, 41, 57]. Examples of push efforts are identifying actionable messages arising from research, fine-tuning the messages for different user groups, working with credible messengers for each group to disseminate the messages, supporting decision making and actions associated with the messages, and developing media releases for the actionable messages, and training researchers to develop their capacity to create, disseminate and execute evidence informed push efforts [25].

What is interesting to note is that most of the areas where policymakers identified a need for improvement fall within the responsibilities of the researchers. Most researchers that focus on health systems and policy research in Israel have acknowledged that they are minimally involved in KTE activities [34]. However, while increasing initiatives both within research production and push efforts to improve the use of HSPR in decision making is important, there are initiatives that can be undertaken by policymakers and decision makers as well.

## Conclusion

This research demonstrated that health systems policymakers in Israel perceive to have strong relationships with researchers; however there is room to improve on these collaborations e.g., partnering in research projects to ensure their relevance. Furthermore, health system policymakers seem to be interested in receiving relevant research in an effective format and are open to using research in decision making. However, providing access to relevant material and assisting target audiences to acquire and use research is rarely done. KTE is a fairly new area in Israel and therefore the level of KTE activities is not very high. Health system and policy researchers in Israel need to be given a deeper understanding of the benefits and potential advantages of KTE in an organized and systematic way and interventions need to be implemented and evaluated to determine the effectiveness in the Israeli context.

## Abbreviations

KTE, Knowledge Transfer and Exchange; HSPR, Health Systems and Policy Research
